# Exploring Multi-Tissue Alternative Splicing and Skeletal Muscle Metabolism Regulation in Obese- and Lean-Type Pigs

**DOI:** 10.3390/genes15020196

**Published:** 2024-01-31

**Authors:** Wei Wang, Wangchang Li, Weiwei Liu, Zishuai Wang, Bingkun Xie, Xiaogan Yang, Zhonglin Tang

**Affiliations:** 1 Key Laboratory of Agricultural Animal Genetics, Breeding and Reproduction of Ministry of Education and Key Lab of Swine Genetics and Breeding of Ministry of Agriculture and Rural Affairs, Huazhong Agricultural University, Wuhan 430070, China; wwlucky1005@163.com; 2 Kunpeng Institute of Modern Agriculture at Foshan, Agricultural Genomics Institute, Chinese Academy of Agricultural Sciences, Foshan 528226, China; liwangchang1019@163.com (W.L.); liuww19990703@163.com (W.L.); wangzishuai@caas.cn (Z.W.); 3 Guangxi Key Laboratory of Animal Breeding, Disease Control and Prevention, College of Animal Science & Technology, Guangxi University, Nanning 530004, China; 4 Shenzhen Branch, Guangdong Laboratory for Lingnan Modern Agriculture, Key Laboratory of Livestock and Poultry Multi-Omics of MARA, Agricultural Genomics Institute at Shenzhen, Chinese Academy of Agricultural Sciences, Shenzhen 518124, China; 5 Animal Husbandry Research Institute, Guangxi Vocational University of Agriculture, Nanning 530001, China; bkxie@163.com

**Keywords:** obese- and lean-type pigs, multiple tissues, transcriptome, alternative splicing, skeletal muscle metabolism

## Abstract

Alternative splicing (AS) is a crucial mechanism in post-transcriptional regulation, contributing significantly to the diversity of the transcriptome and proteome. In this study, we performed a comprehensive AS profile in nine tissues obtained from Duroc (lean-type) and Luchuan (obese-type) pigs. Notably, 94,990 AS events from 14,393 genes were identified. Among these AS events, it was observed that 80% belonged to the skipped exon (SE) type. Functional enrichment analysis showed that genes with more than ten AS events were closely associated with tissue-specific functions. Additionally, the analysis of overlap between differentially alternative splicing genes (DSGs) and differentially expressed genes (DEGs) revealed the highest number of overlapped genes in the heart and skeletal muscle. The novelty of our study is that it identified and validated three genes (*PYGM*, *MAPK11* and *CAMK2B*) in the glucagon signaling pathway, and their alternative splicing differences were highly significant across two pig breeds. In conclusion, our study offers novel insights into the molecular regulation of diverse tissue physiologies and the phenotypic differences between obese- and lean-type pigs, which are helpful for pig breeding.

## 1. Introduction

Alternative splicing (AS) is a prevalent and evolutionarily conserved biological process in which splice sites are differentially selected within pre-messenger RNAs, leading to the generation of diverse mRNA and protein isoforms [1,2,3]. The growing body of evidence suggests that the precise regulation of AS plays a crucial role in determining tissue types and developmental stages [4]. Researchers have discovered that transcripts from ~95% of multi-exon genes undergo alternative splicing in humans [5]. Meanwhile, disruptions in the splicing pathway or the presence of aberrant splicing isoforms have been associated with various human diseases [6,7]. And five different types of alternative splicing events have been identified, including skipped exons (SEs), retained introns (RIs), alternative 5′ splice sites (A5SSs), alternative 3′ splice sites (A3SSs), and mutually exclusive exons (MXEs) [8,9].

Pigs are an important source of animal protein for humans [10]. They are also an ideal medical model for many biomedical research disciplines because of their similarity to humans in size, immunology, anatomy, genome, and physiological characteristics [11,12,13]. There are two pig breeds, Western pigs (lean-type) and Chinese native pigs (obese-type), and their phenotypes exhibit significant differences [14]. For example, the former is characterized by more developed muscles, smaller muscle fibers, higher muscle content, and lower fat content in their muscles. Conversely, the latter exhibit higher levels of fat deposition, including subcutaneous, visceral, and intermuscular fat [15]. In previous studies, significant differences in gene expression and mutations have been found to partially explain the variations between breeds with different phenotypes [16,17,18]. Due to the aforementioned differences, these two breeds become valuable resources for the study of AS. While there has been extensive exploration of AS events across various tissues in pigs [19,20], the differences in AS events within multiple tissues of lean-type and obese-type pig breeds, as well as the regulatory functions of different transcripts, remain not fully understood.

In the present study, we conducted a comprehensive comparative analysis of the AS characteristics in nine tissues (liver, lung, skeletal muscle, heart, adipose, cerebrum, cerebellum, stomach, and small intestine) from Duroc (DR) and Luchuan (LC) pigs. The results revealed the detection of 94,990 AS events, of which 87% were novel, originating from 14,393 genes across these tissues. Subsequently, we analyzed differentially alternative splicing genes (DSGs) in various tissues and pig breeds, with a particular focus on exploring DSGs associated with skeletal muscle metabolism. We aimed to explore AS in pigs, and the data not only enhance our understanding of pig gene functionality, including the distinct functions of different gene isoforms and regulatory mechanisms, but also provide important resources for pig genetic breeding.

## 2. Materials and Methods

### 2.1. Data Collection

The transcriptome datasets of nine tissues (liver, lung, skeletal muscle, heart, adipose, cerebrum, cerebellum, stomach, and small intestine) from DR and LC pigs were obtained from our previous study [21] and are available at the China National GenBank (https://db.cngb.org/search/project/CNP0001159/ (accessed on 5 December 2023)) Nucleotide Sequence Archive (CNSA) under the accession number CNP0001159. Briefly, six adult individuals for DR and LC pigs (three sows for each breed) were obtained from the Institute of Animal Science, Guangxi Zhuang autonomous Region, China. Nine tissues or organs were collected from each pig, resulting in a total of 54 transcriptomes sequenced. The accession numbers for each sample are listed in Table S1.

### 2.2. Identification and Quantification of Transcripts

Quality control for the raw reads was performed using Fastp software version 0.23.1 with the default parameters [22]. High-quality reads were aligned to the Sus Scrofa 11.1 reference genome using Hisat (version 0.1.6) with the default parameters [23,24]. Only reads uniquely aligned to the reference genome were used for downstream analysis. Transcript construction for each sample was performed using StringTie (v1.3.3) with default parameters. The abundance of the transcripts was measured by TPM (Transcripts Per Million). Transcripts with TPM lower than 0.1 were filtered out [25]. Identification of known transcripts and novel transcripts was performed by comparing with the Sus Scrofa 11.1 genome annotation file (Ensembl release 108) using StringTie. Functional enrichment analyses of overlapping genes were performed using DAVID version 6.8 [26], enabling Gene Ontology (GO) terminology and the Kyoto Encyclopedia of Genes and Genomes (KEGG) pathway function. False discovery rate (FDR) values ≤0.05 were considered to be a significant function.

### 2.3. Analysis of Differentially Expressed Genes, Transcripts and Alternative Splicing

Differentially expressed genes (DEGs) and differentially expressed transcripts (DETs) were identified via the Deseq2 version 1.36.0 R package [27]. Genes with a false discovery rate (FDR) ≤ 0.05 and |log2FoldChange| ≥ 1 were considered as DEGs. Transcripts with a false discovery rate (FDR) ≤ 0.05 and |log2FoldChange| ≥ 1 were considered as DETs.

The rMATS program was used to classify and count the five types of alternative splicing events. The differences in AS events between DR and LC pigs were measured by variation in percentage spliced in (PSI), a commonly used parameter to describe the degree of alternative splicing in international biomedical research organizations. The estimated value of PSI is shown in the following equation:

$$\mathrm{PSI}=\frac{R_{\mathrm{Splice}\_\mathrm{in}}}{R_{\mathrm{Splice}\_\mathrm{in}}+R_{\mathrm{Splice}\_\mathrm{out}}}\;\Delta \mathrm{PSI}=\mathrm{PS}I_{DR}-\mathrm{PS}I_{\mathrm{LC}}$$

$R_{\mathrm{Splice}\_\mathrm{in}}$ represents the count of reads specific to the splicing transcript, and $R_{\mathrm{Splice}\_\mathrm{out}}$ represents the count of reads specific to the no-splicing transcript. ${\mathrm{PSI}}_{\mathrm{DR}}$ represents the percent spliced in DR pigs. ${\mathrm{PSI}}_{\mathrm{LC}}$ represents the percent spliced in LC pigs. ΔPSI means the value of change.

Alternative splicing events with FDR ≤ 0.05 and |ΔPSI| ≥ 10% were extracted as differential alternative splicing events.

### 2.4. RNA Extractison

Total RNA was extracted from quick-frozen samples using TRIzol^®^ reagent (Invitrogen Life Technologies, Palo Alto, CA, USA) according to the manufacturer′s instructions. The RNA quality was determined using agarose gel electrophoresis and NanoDrop 2000 (Thermo Fisher Scientific, Waltham, MA, USA).

### 2.5. Semi-Quantitative RT-PCR Analysis of Alternative Splicing Events

Primers flanking the differentially spliced region were designed using the Primer Premier 5. The primers are shown in Table S2. PCR products were separated by 2% agarose gel in 1×TAE buffer for 40–60 min at 120 V. ImageJ software (v.1.47) was used to define regions of interest (ROIs) [28,29]. Independent measurements of the surveyed area were made using ROIs of identical size. DNA fragments were recovered from the gel, followed by Sanger sequencing at the Sangon Biotech company (Shanghai, China).

### 2.6. Statistical Analysis

GraphPad Prism v8.0.2 was used for statistical analysis. An unpaired nonparametric test for statistical significance was performed. The significance level was set at *p*-value < 0.05. Data were expressed as mean ± standard error of the mean (SEM) (n = 3).

## 3. Results

### 3.1. Tissue- and Breed-Specific Differential Expression Profiles of Lean- and Obese-Type Pigs

**Figure 1:**
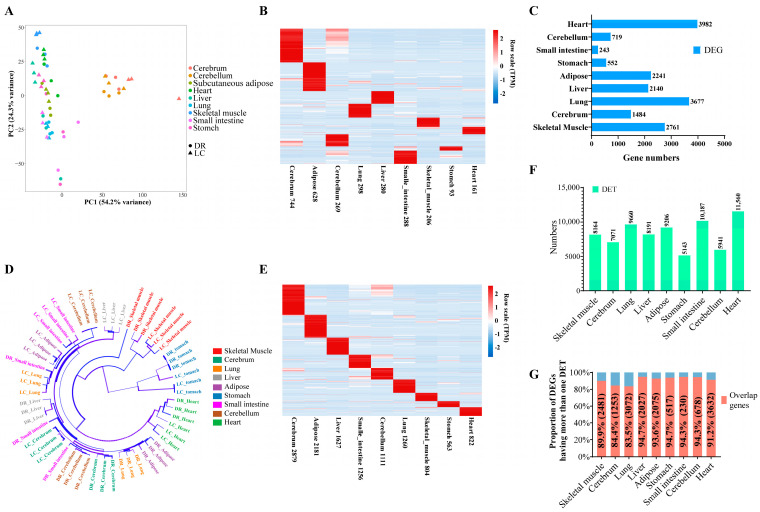
The expression characteristics of genes and transcripts in different tissues and breeds. (**A**) Principal coordinate analysis of nine tissues in DR and LC pigs. Colors: different tissues. Shape: different breeds. (**B**) Heatmap of tissue-specific genes among different tissues. (**C**) The DEGs in nine tissues between DR and LC pigs. (**D**) Hierarchical clustering tree of nine tissues in DR and LC pigs. Colors: different tissues. (**E**) Heatmap of tissue-specific expressed transcripts in different tissues. (**F**) The DETs in nine tissues between DR and LC pigs. (**G**) The proportion of differentially expressed genes with at least one differentially expressed transcript. DR: Duroc pigs. LC: Luchuan pigs. DEGs: differentially expressed genes. DETs: differentially expressed transcripts.

The RNA sequencing data exhibited high quality, with an average of 45.79 million high-quality clean reads, revealing a high average percentage of 96.90% and 92.20% for Q20 and Q30, respectively (Table S3). The quantification information for identified genes and transcripts is provided in Table S4 and Table S5, respectively. A PCA was conducted to analyze the gene expression patterns of different tissues and biological replicates from DR and LC pigs (Figure 1A). The results demonstrated distinct clustering patterns among tissues and biological replicates, with notable differences in clustering observed among tissues from the two breeds. Then, we used TBtools (v2.041) to identify the number of tissue-specific expressed genes in these nine tissues. As we can see, the highest and lowest number of tissue-specific expressed genes were found in the cerebrum (744) and stomach (93) (Figure 1B). Additionally, in a more focused investigation on pig breeding, the number of tissue-specific expressed genes was 206 in skeletal muscle and 628 in adipose tissue (Figure 1B). Meanwhile, the heart exhibited the highest number of differentially expressed genes (DEGs) between DR and LC pigs (Figure 1C). Moreover, there were also numerous differentially expressed genes in skeletal muscle (2761) and adipose tissue (2241) (Figure 1C).

Currently, an increasing amount of research on gene function is directing attention to the regulatory functional aspects of different gene isoforms. Therefore, we conducted an exploration of tissue-specific and breed-specific transcripts. Hierarchical clustering based on expression levels of transcript also showed clear separations among different tissues (Figure 1D). The analysis of tissue-specific transcripts showed that the cerebrum had the highest number of transcripts (Figure 1E). Notably, the heart and stomach had the highest and lowest number of differentially expressed transcripts (DETs) between DR and LC pigs, respectively (Figure 1F). The analysis of tissue-specific transcripts provided additional insights into the complex regulatory mechanism of gene expression. We found that at least 83.5% of DEGs had at least one DET (Figure 1G), suggesting that differential gene expression might be caused by the differential expression of transcripts [30]. These findings shed light on the intricate regulatory mechanisms that govern differential gene expression in different pig tissues and breeds. Simultaneously, we have identified numerous tissue- and breed-specific functional genes or transcripts, which can be regarded as potential candidate genes (transcripts) for regulating phenotypic differences between lean- and obese-type pigs.

### 3.2. Five Types of Alternative Splicing Events Generate Novel Transcripts

**Figure 2:**
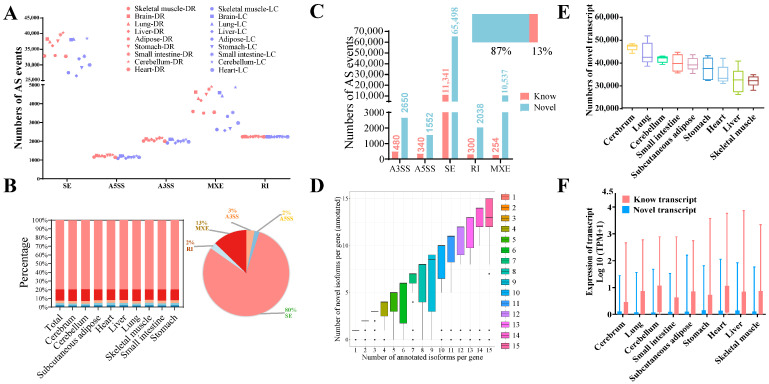
Analysis of alternative splicing and identification of novel transcripts in nine tissues between two breeds. (**A**) Number of AS events in each sample from nine tissues in DR and LC pigs. Colors: different tissues. Shape: different breeds. (**B**) The distribution of five types of AS events in nine tissues. Left: proportion of five AS events in nine tissues. Right: total percentage of five AS events. (**C**) The number of known and novel AS events. Top right: percentage of novel AS events. (**D**) The relationship between the number of annotated isoforms and novel isoforms per gene. (**E**) The distribution of novel transcripts identified in nine different tissues (TPM > 0.1). (**F**) Expression abundance of novel and known transcripts (log2(TPM + 1) in nine different tissues. AS: alternative splicing. A3SS: alternative 3′ splice sites, A5SS: alternative 5′ splice sites, MXE: mutually exclusive exons, RI: retained intron, SE: skipped exon. TPM: Transcripts Per Million.

We found that the number of AS events ranged from 35,377 to 48,517 in nine tissues from two pig breeds. These AS events were divided into five types, including skipped exons (SE), retained introns (RIs), alternative 5′ splicing sites (A5SSs), alternative 3′ splicing sites (A3SSs), and mutually exclusive exons (MXEs). The statistical results indicated that the number of SE-type and MXE-type events ranked first and second, respectively, while the number of A5SS-type events was the least (Figure 2A). To be specific to individual tissues, there was a similar distribution of the AS type across nine different tissues in two breeds (Figure 2B, left panel). Above all, 80% of them belonged to the SE type (Figure 2B, right panel).

The novel AS events were categorized into two types, novel transcripts of known genes and novel transcripts of unknown genes. In our study, we found that more than 87% of AS events were novel transcripts (Figure 2C). The correlation between the number of previously annotated isoforms and novel isoforms of annotated genes was analyzed. Transcripts with ensemble IDs were considered to be annotated: the greater the number of annotated isoforms per gene, the greater the number of novel isoforms detected (Figure 2D). The cerebrum and lung had a higher number of novel transcripts compared to the other tissues analyzed, and to our surprise, the skeletal muscle had a lower number of novel transcripts (Figure 2E). This might indicate that the genetic activity in the skeletal muscle is relatively more well studied or understood compared to the cerebrum and lung in pigs. Due to the observation of a higher number of novel transcripts compared to known transcripts, we aimed to explore the expression levels of these novel transcripts. However, it should be noted that, despite the considerably lower expression level of novel transcripts compared to known transcripts (Figure 2F), it was still worthwhile to explore their contribution to gene expression and their impact on gene function. These findings suggest that AS is a highly complex regulatory mechanism in gene expression across various tissues, with the SE type being the most prevalent AS event in all tissues. Additionally, new AS events have been identified, and the corresponding novel transcripts generated require further identification and exploration.

### 3.3. Tissue-Specific Alternative Splicing Events

**Figure 3:**
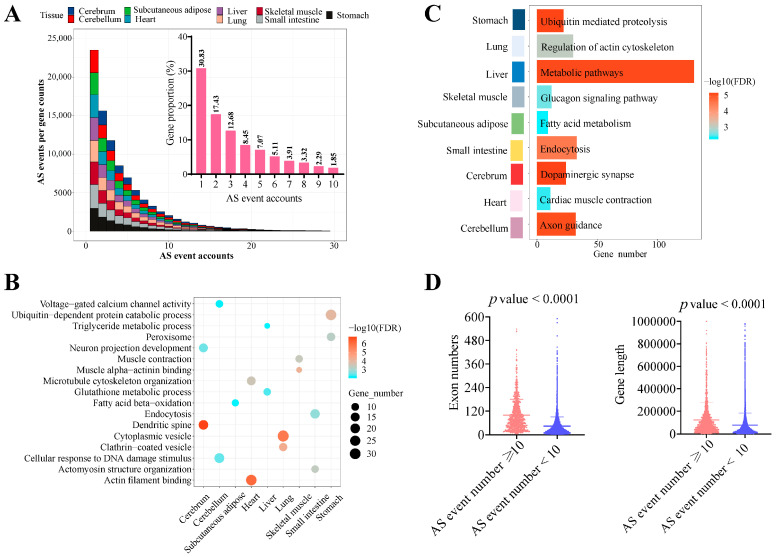
Molecular characteristics of tissue-specific alternative splicing events. (**A**) Distribution of AS events produced from genes in different tissues. Colors: different tissues. Top right: proportion of AS events among genes. (**B**) GO analysis for genes with more than ten AS events. (**C**) The analysis of KEGG pathways for genes with more than ten AS events. (**D**) The exon number and sequence length for genes with AS events. GO: Gene Ontology. KEGG: Kyoto Encyclopedia of Genes and Genomes.

Previous studies have demonstrated a positive correlation between the number of alternative splicing (AS) events and the number of transcripts produced by a gene [31,32]. In our study, we observed a decreasing trend in the number of AS events per gene as the range of AS events increased from 1 to 30 (Figure 3A, Table S6). Specifically, we found that genes with only one AS event comprised the highest proportion, accounting for an average of approximately 30.83%. Furthermore, nearly 93% of all genes had fewer than ten AS events in pigs. Therefore, for genes with more than ten AS events, accounting for less than 10%, we were curious about the main regulatory pathways these genes were involved in. So, we performed functional enrichment analysis on genes with more than ten AS events to elucidate the logic of AS event variation. GO analysis revealed that these genes were associated with tissue-specific functions (Figure 3B), such as muscle contraction in skeletal muscle. KEGG pathway analysis showed enrichment of metabolic pathways specific to each tissue, including metabolic pathways in the liver, fatty acid metabolism in subcutaneous adipose tissue, and the glucagon signaling pathway in skeletal muscle (Figure 3C). Additionally, genes with more than ten AS events had more exons and were significantly longer (Figure 3D). These findings suggest that genes involved in AS events are closely associated with tissue-specific functions, and genes with more than ten AS events are predominantly involved in metabolic pathways.

### 3.4. Comparison Analysis of Alternative Splicing between Duroc and Luchuan Pigs

**Figure 4:**
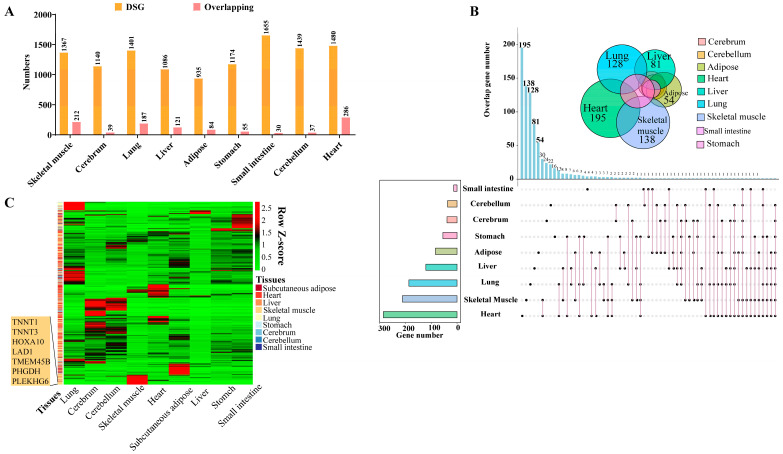
Comparison analysis of AS events in different tissues between DR and LC pigs. (**A**) Statistical analysis of DSGs and overlapping genes in nine tissues between two breeds. (**B**) Distribution of the number of overlapping genes and the number of tissue-specific AS genes in different tissues. (**C**) Expression pattern of tissue-specific AS genes in nine tissues. Left: genes specific to skeletal muscle.

Differentially alternative splicing genes (DSGs) were identified between DR and LC pigs in the same tissues. We found that the number of DSGs ranged from 935 to 1655; these were the lowest and highest numbers of DSGs in subcutaneous adipose and small intestine between the two breeds, respectively. In skeletal muscle and heart tissues, the numbers of DSGs between DR and LC pigs were 1367 and 1480. Furthermore, the overlap analysis between DSGs and differentially expressed genes (DEGs) showed that the heart and skeletal muscle had the highest number of shared genes (Figure 4A). Meanwhile, the KEGG and GO analyses of the overlapping genes revealed significant differences among the different tissues (Figures S1 and S2). For example, we found that the heart exhibits tissue-specific regulation of cell cycle function, while the liver exhibits tissue-specific regulation of cell proliferation function, and the lung exhibits tissue-specific regulation of cell shape function [33,34,35]. These findings suggest that different tissues have evolved specialized molecular mechanisms to regulate specific biological functions.

To gain further insights into tissue-specific AS gene expression, we performed a multi-tissue overlapping analysis. The results showed that the heart (195), skeletal muscle (138) and lung (128) exhibited a large number of tissue-specific AS genes (Figure 4B). The further analysis suggested that several genes, such as *TNNT1* (troponin T1, slow skeletal type), *TNNT3* (troponin T3, fast skeletal type), *HOXA10* (Homeobox A10), *LAD1* (Ladinin 1), *TMEM45B* (Transmembrane protein 45B), *PHGDH* (phosphoglycerate dehydrogenase) and *PLEKHG6* (Pleckstrin Homology and RhoGEF Domain Containing G6), present the highest tissue-specific AS in skeletal muscle (Figure 4C), indicating their involvement in maintaining intramuscular homeostasis and muscle development. These findings indicated that tissue-specific AS genes play a crucial role in the growth, development and maintenance of tissues. Furthermore, we identified some AS genes that are specifically expressed in skeletal muscle. These genes may serve as potential functional candidates regulating the phenotypic differences in skeletal muscle between lean- and obese-type pigs.

### 3.5. AS Analysis of Genes from Glucagon Signaling Pathway in Skeletal Muscle

**Figure 5:**
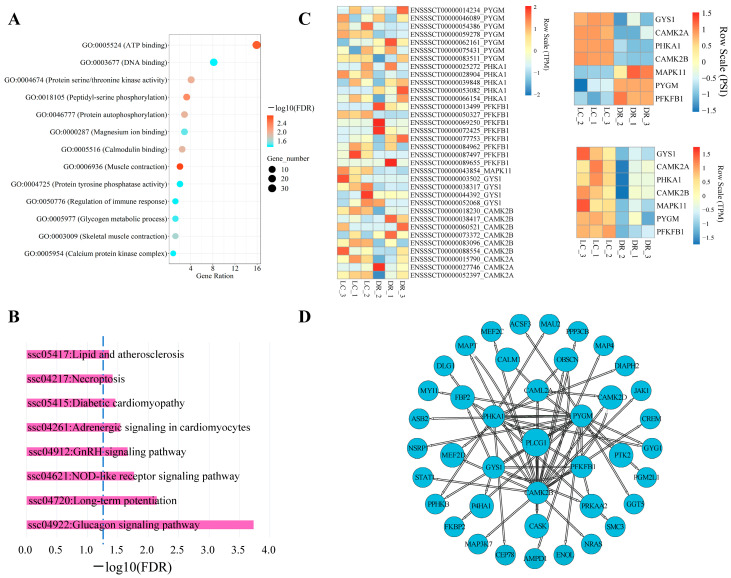
The differential analysis of alternative splicing events in skeletal muscle between Duroc and Luchuan pigs. (**A**) GO analysis of specific AS genes in skeletal muscle. (**B**) KEGG analysis of specific AS genes in skeletal muscle. (**C**) The differential expression of AS genes from the glucagon signaling pathway in skeletal muscle between DR and LC pigs. The left panel shows the transcripts expression patterns, the upper right panel shows the AS events expression patterns, and the lower right panel shows the genes expression patterns. (**D**) Interaction networks of genes from the glucagon signaling pathway. TPM: Transcript per Million. PSI: percentage spliced in. DR: Duroc pigs. LC: Luchuan pigs.

Skeletal muscle constitutes ~40% of body mass and has the capacity to play a major role as thermogenic, metabolic and endocrine tissue [36]. In our study, GO analysis revealed that differentially alternative splicing genes (DSGs) between DR and LC pigs in skeletal muscle were enriched in the muscle contraction response process and glycogen metabolic process (Figure 5A). Moreover, KEGG analysis demonstrated that these DSGs were significantly enriched in the glucagon signaling pathway (FDR = 1.85 × 10^−4^) in both DR and LC pigs (Figure 5B, Table S7).

Subsequently, we further focused on the genes related to glucagon signaling pathway, including *PFKFB11* (6-phosphofructo-2-kinase/fructose-2,6-biphosphatase), *MAPK11* (mitogen-activated protein kinase 11), *PYGM* (glycogen phosphorylase, muscle-associated), *CAMK2B* (calcium/calmodulin-dependent protein kinase II β), *CAMK2A* (calcium/calmodulin-dependent protein kinase II α), GYS1 (glycogen synthase 1) and *PHKA1* (phosphorylase kinase regulatory subunit α 1). We analyzed the expression differences of differential splicing events, differential transcripts and differential genes between DR and LC pigs. We found that the PSI values of *GYS1*, *CAMK2A*, *PHKA1* and *CAMK2B* were higher in DR pigs compared to LC pigs, Furthermore, the PSI values of *MAPK11*, *PYGM* and *PFKFB1* had the opposite trend between DR and LC pigs (Figure 5C, Top right). Due to the occurrence of AS events leading to the generation of distinct mRNA isoforms, we conducted an examination of the expression differences in various transcripts of the aforementioned genes in DR and LC pigs (Figure 5C, Left panel). Furthermore, these transcripts differences will ultimately lead to variations in the overall gene expression levels (Figure 5C, Bottom right). The results highlight the expression levels of all these genes were significantly different at both the transcript and gene level between two breeds.

To investigate the relationships between alternative splicing genes, we calculated the correlation between their expression levels (TPM) and identified correlations greater than 0.85 as being relevant. A correlation network showed that *PYGM*, *PFKFB1*, *CAMK2B*, *GYS1*, *PHKA1* and *CAMK2A* may play important roles in regulating these genes (Figure 5D). These results suggested that the AS of these genes related to the glucagon signaling pathway significantly contributes to the phenotype differences of skeletal muscle between the two breeds.

### 3.6. Validation of AS Differences of Genes Related to the Glucagon Signaling Pathway

**Figure 6:**
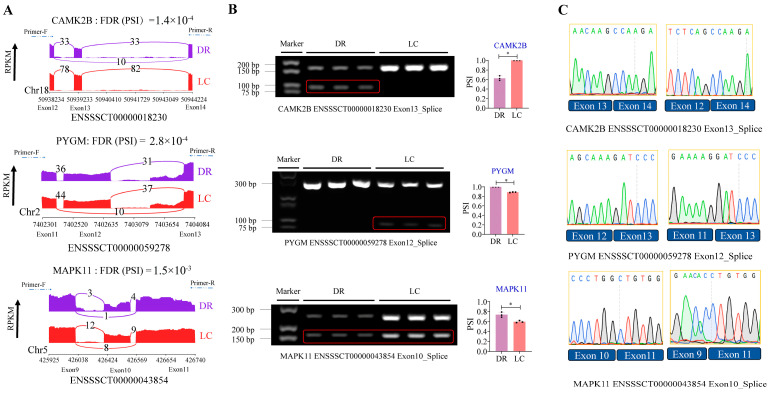
Transcript information of the novel isoforms and verification of alternative splicing events (*CAMK2B*, *PYGM* and *MAPK11*). (**A**) RNA-seq results of AS using sashimi plot analysis. Read count for standardized-RPKM. Arrows of the primers represent the amplification of three exonic regions. (**B**) Semi-quantitative RT-PCR analyses for AS genes between DR and LC pigs. Left: exon skipping event verification. Right: verification of different AS event data (PSI). Data are presented as mean ± standard error of the mean (SEM), n = 3. * *p* < 0.05. (**C**) Sanger sequencing results of splicing sites for different transcripts of genes. PSI: percentage spliced in. RPKM: Reads Per Kilobase per Million mapped reads.

To confirm the results of the AS analysis, we selected three genes (*CAMK2B*, *PYGM*, and *MAPK11*) related to the glucagon signaling pathway to validate differences in AS events in skeletal muscle between DR and LC pigs. The sashimi plot results display the exon positions of *CAMK2B*, *PYGM* and *MAPK11* undergoing alternative splicing, along with the corresponding reads count. The primer positions used for semi-quantitative detection were also marked. It can be observed that in the LC pigs, there was no skipping of exon 13 in *CAMK2B*, while in the DR pigs, there was no splicing of exon 12 in *PYGM* (Figure 6A, Table S8). The semi-quantitative RT-PCR experiments validated the alternative splicing of these three genes in DR and LC pigs, confirming consistency with the results shown in the sashimi plot. PSI values of *PYGM* and *MAPK11* were significantly higher in DR pigs, while PSI values of *CAMK2B* were lower in DR pigs (Figure 6B), which were consistent with the analysis results based on RNA-sequencing data. Subsequently, we subjected the products of semi-quantitative RT-PCR to Sanger sequencing and conducted nucleic acid sequence alignment between DR and LC pigs. The results indicated that, indeed, exon skipping occurred in exon 13 of *CAMK2B*, exon 12 of *PYGM*, and exon 10 of *MAPK11* (Figure 6C). These results confirm the reliability of our bioinformatics analysis of AS events and simultaneously unveil differentially spliced genes in skeletal muscle between DR and LC pigs.

## 4. Discussion

Transcriptome analysis provides an excellent method for understanding the molecular characteristics of the physiological function of an organ or tissue [17,37,38,39]. A comprehensive analysis of AS (alternative splicing)-mediated gene expression changes in pig tissues is essential for advancing pig breeding [40]. pre-mRNAs perform distinct regulatory functions in specific tissues through different AS types [41]. In this study, we systematically analyzed AS in nine tissues from DR (lean-type) and LC (obese-type) pigs (Figure S3). A total of 94,990 AS events and 14,393 expressed genes were detected, with an average of 6.6 AS events per gene. However, other related studies have reported an average of 2.3 AS events per gene [42]. The observed difference in the number of AS events per gene may be attributed to breed and tissue-specific factors. Furthermore, our study found that SE events were the most common forms of AS, consistent with previous research findings [43,44]. However, there is also a study that has found SE events not to be the most prevalent type among AS events [31]. Moreover, unlike in animals, retained intron (RI) is the most conspicuous type of alternative splicing in plants [45]. In comparison to annotated isoforms, our study revealed a significant increase in novel isoforms and transcripts, whereas some studies have indicated no differences [46]. This discrepancy could be attributed to inconsistencies in genome assembly (Sus Scrofa 11.1 vs Sus Scrofa 10.2) and annotation methods. In conclusion, these novel transcripts and isoforms provide important insights into pig genome annotation. However, it is worth noting that novel transcripts generated by AS may undergo nonsense-mediated decay (NMD), and NMD plays a role in quality control for the AS process [47,48].

The additional analysis we conducted on the characteristics of tissue-specific AS events in various pig tissues revealed that AS plays a critical role in maintaining specific functions in particular tissue types. Our findings demonstrated a substantial variation in the number of differential alternative splicing genes (DSGs) and differentially expressed genes (DEGs) across different tissues (Table S9). Through overlapping analysis of DSGs and DEGs, we found that the skeletal muscle and heart exhibited a higher number of overlapping genes compared to other tissues. This may be attributed to the presence of some muscle-specific splicing factors in muscle tissue [49,50,51]. Previous studies have also indicated significant differences in AS events between lean and obese-type pigs in skeletal muscle [52]. Specifically, there were numerous AS events related to mitochondrial function and glucose metabolism in the skeletal muscle of lean-type pigs. In contrast, the skeletal muscle of obese-type pigs exhibits abundant AS events related to muscle development and fat metabolism. In the present study, the average expression levels of certain AS genes (e.g., *TNNT1*, *TNNT3*, *LAD1*, *TMEM45B*, *PHGDH* and *PLEKHG6*) were higher in skeletal muscle than in other tissues (Figure 4C). And, based on our knowledge, we identified for the first time that genes such as *TNNT1*, *TNNT3*, and *PHGDH* are skeletal muscle-specific alternative splicing genes, and they exhibit the highest expression levels among all differentially alternative splicing genes between lean-type and obese-type pigs. Based on existing studies, we know that *TNNT1*, *TNNT3* [53,54,55,56,57], and *PHGDH* genes [58,59,60] play important roles in muscle development and metabolic regulation. Therefore, these skeletal muscle-specific DSGs may be related to muscle metabolism and development differences, potentially influencing the formation of lean- and obese-type pigs.

Skeletal muscle is the dominant organ system in locomotion and energy metabolism [61,62]. To further investigate the pathways enriched by DSGs in skeletal muscle between the two pig breeds, GO and KEGG analysis were employed. The results showed that the most significant enrichment pathway was the glucagon signaling pathway. Through our investigation, we focused on three genes associated with skeletal muscle development in this pathway: *PYGM* [63], *MAPK11* [64] and *CAMK2B* [65]. As demonstrated by our results (Figure 6), the PSI values for these three genes showed significant differences in skeletal muscle of two pig breeds. In the semi-quantitative RT-PCR results, it was evident that a transcript of the *CAMK2B* (ENSSSCT00000018230) was not detected in Luchuan pigs. Simultaneously, a transcript of the *PYGM* (ENSSSCT00000059278) was not detected in Duroc pigs. Due to the increasing focus on the diverse regulatory functions exhibited by different protein isoforms encoded by the same gene [66,67,68,69], we contemplate whether the specific transcripts of these three genes contribute to the phenotypic differences observed in skeletal muscles between the two pig breeds.

Our study revealed a significant number of tissue-specific alternative splicing events, with the identified exons serving as potential gene-editing sites. Drawing on the mechanism of exon skipping, these exons can be excised at the mRNA level through precise mutations at conserved bases in splice donors or acceptors [70,71]. This strategy has proven effective in repairing the coding sequence of the DMD causative gene in animal models [72] and also provides a new method for *MSTN* gene knockout in pigs [73]. An interesting observation in our study is that, consistent with the majority of alternative splicing classifications [2,74,75], we divided AS events into five types. However, in some analyses, AS events were categorized into seven classes, including an additional two events: alternative first exon (AF) and alternative last exon (AL) events [76,77,78,79]. Additionally, upon consideration, we can specifically tally the number of microexons within SE events when conducting alternative splicing analysis, for instance, counting the microexons involved in differential SE events between the two pig breeds. Through transcriptomic analyses of several species and organs, microexons were found to have the highest evolutionary conservation of sequence and inclusion levels relative to other classes of alternative splicing elements [3,80]. Finally, in our study, the fusion transcripts, full-length mRNAs and APA (alternative polyadenylation) sites [81] of two pig breeds have not been well characterized due to the lack of full-length transcripts, so single-molecule long-read sequencing (PacBio Iso-seq) can be used to directly obtain full-length transcripts without further assembly, thus overcoming the above-mentioned limitations [76,82,83,84]. The strategy of combining Iso-seq and RNA-seq techniques can improve the efficiency of pig genome annotation [85].

## 5. Conclusions

Our study conducted comprehensive alternative splicing profiling in nine tissues from two pig breeds (Duroc and Luchuan) with different traits (lean- and obese-type). We identified tissue-specific differentially expressed genes and differentially alternative splicing genes across the different tissues and breeds, and validated the DSGs related to the glucagon signaling pathway in the skeletal muscle of two pig breeds. These data can be valuable for elucidating the transcriptomic profile and enhancing the genome annotation of pigs, providing helpful insights for pig breeding.

## Data Availability

All the relevant data are provided along with the manuscript as Supplementary Files.

## Supplementary Materials

The following supporting information can be downloaded at: https://www.mdpi.com/article/10.3390/genes15020196/s1, Table S1: Transcriptome data acquisition table for 54 samples; Table S2: Primer details; Table S3: Alignment information of nine tissues; Table S4: The identification and quantification information of gene; Table S5: The identification and quantification information of transcript; Table S6: Genes with ten or more alternative splicing events across the nine tissues; Table S7: The KEGG pathways enriched by skeletal muscle-specific DSGs; Table S8: Alternative splicing events for three genes; Table S9: Statistical analysis of the number of DEGs and DSGs; Figure S1: Bubble chart depicting enriched KEGG pathway analysis of DEGs of DSGs in nine tissues; Figure S2: Bubble chart depicting enriched GO function analysis DEGs of DSGs in nine tissues; Figure S3: Graphical abstract showing RNA-seq data collection and experimental design for analysis in Luchuan and Duroc pigs.
