# Supplementary figures and images for: Exploring Multi-Tissue Alternative Splicing and Skeletal Muscle Metabolism Regulation in Obese- and Lean-Type Pigs
Source: Genes (Basel). 2024 Jan 31;15(2):196. doi: 10.3390/genes15020196 (PMC10888101; doi:10.3390/genes15020196)

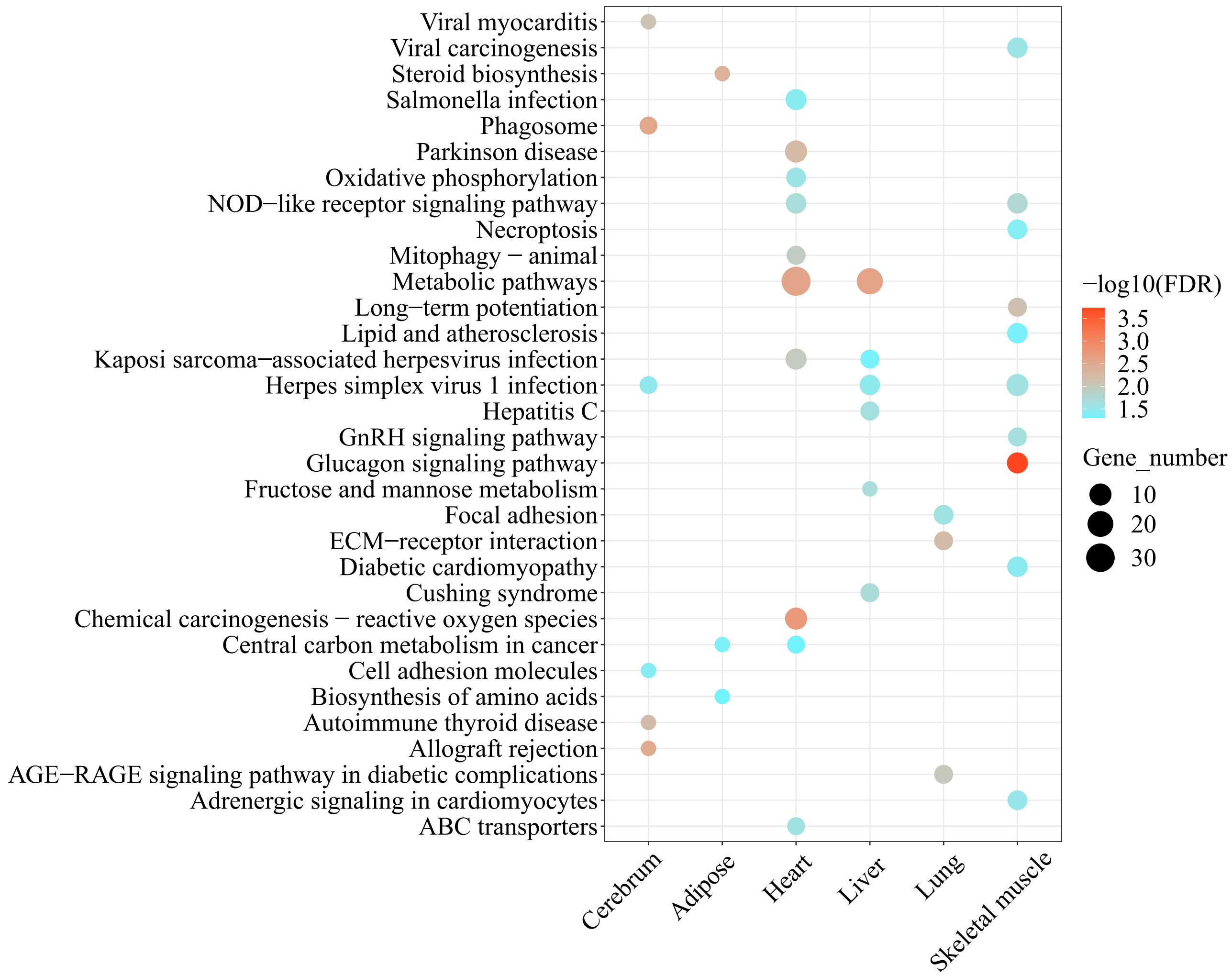

Supplement: Supplementary file 1 [file genes-15-00196-s001.zip › Figure S1.pdf]

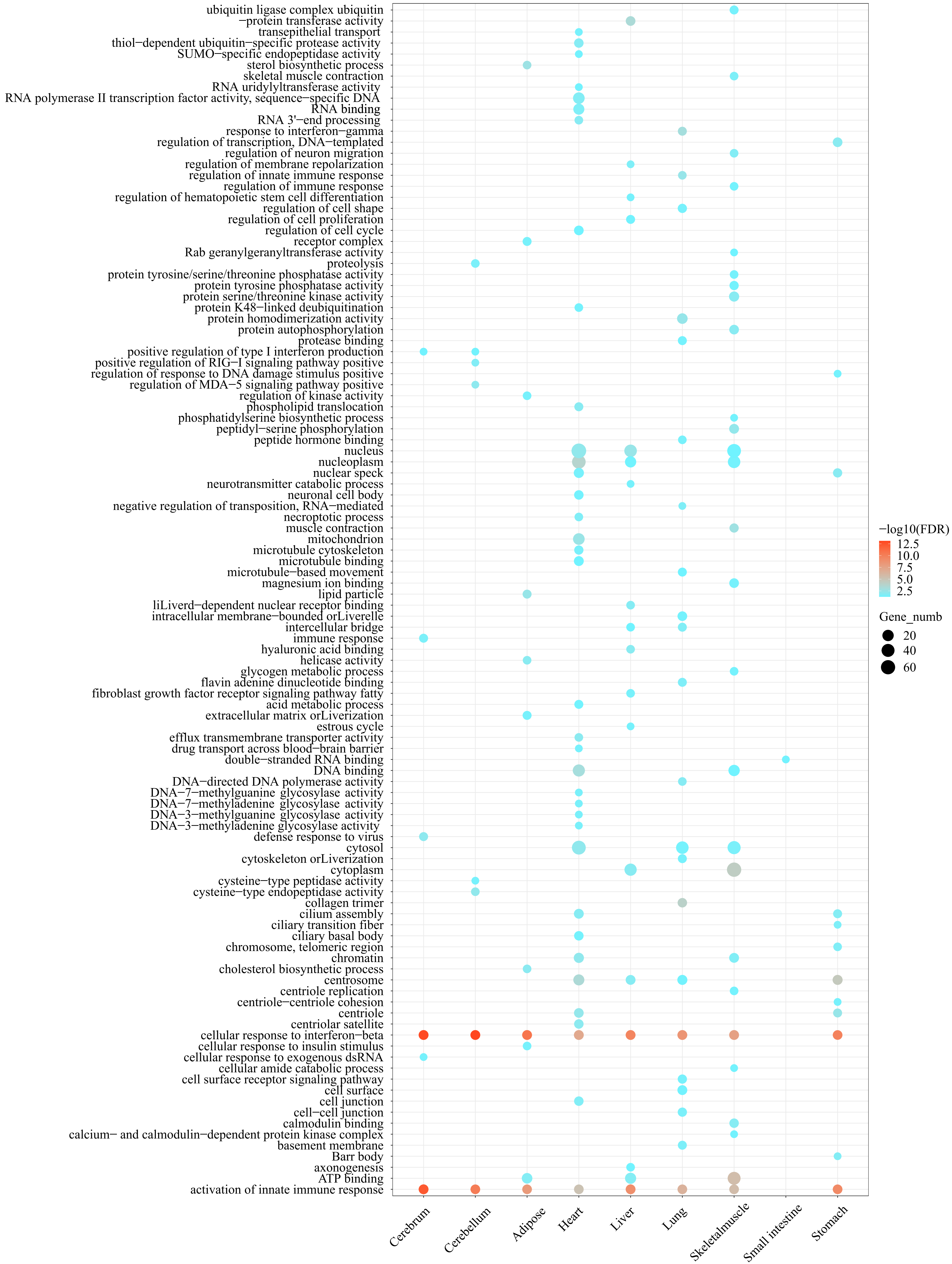

Supplement: Supplementary file 1 [file genes-15-00196-s001.zip › Figure S2.pdf]

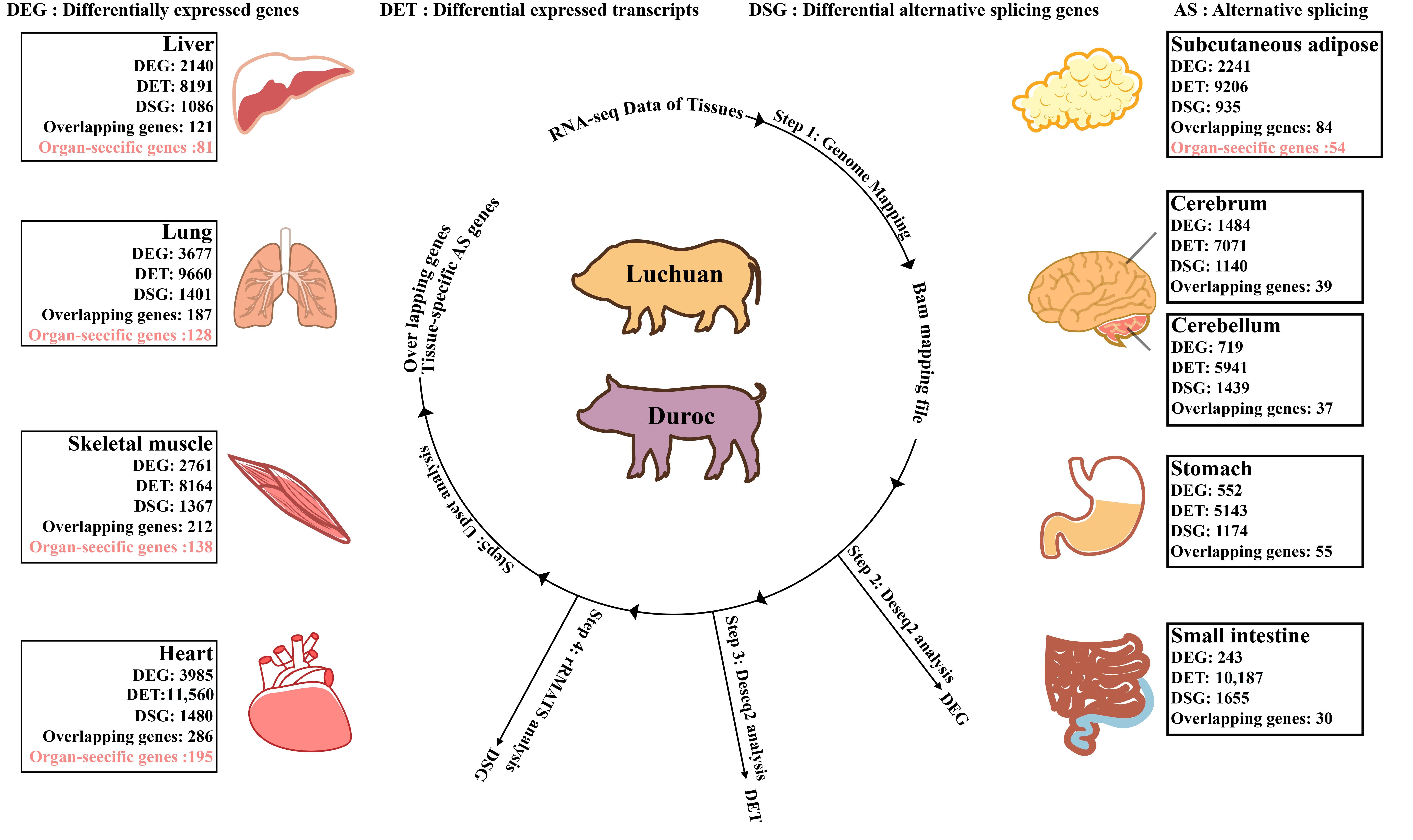

Supplement: Supplementary file 1 [file genes-15-00196-s001.zip › Figure S3.jpg]
